# Synthesis and Anchoring of Antineoplastic Ferrocene and Phthalocyanine Derivatives on Water-Soluble Polymeric Drug Carriers Derived from Lysine and Aspartic Acid

**DOI:** 10.1155/2008/217573

**Published:** 2007-12-06

**Authors:** M. David Maree, Eberhard W. Neuse, Elizabeth Erasmus, Jannie C. Swarts

**Affiliations:** ^1^Department of Chemistry, University of the Free State, Bloemfontein 9300, South Africa; ^2^School of Chemistry, University of the Witwatersrand, PO WITS 2050, South Africa

## Abstract

The general synthetic strategy towards water-soluble biodegradable drug carriers and the properties that they must have are discussed. The syntheses of water-soluble biodegradable copolymers of lysine and aspartic acid as potential drug-delivering devices, having amine-functionalised side chains are then described. Covalent anchoring of carboxylic acid derivatives of the antineoplastic ferrocene and photodynamically active phthalocyanine moieties to the amine-containing drug carrier copolymers under mild coupling conditions has been achieved utilising the coupling reagent O-benzotriazolyl-N,N,N^′^,N^′^-tetramethyluronium hexafluorophosphate to promote formation of the biodegradable amide bond. Even though the parent antineoplastic ferrocene and phthalocyanine derivatives are themselves insoluble in water at pH < 7, the new carrier-drug conjugates that were obtained are well water-soluble.

## 1. INTRODUCTION

In accordance with the goals of this special issue of metal-based drugs, this article deems to fulfil a teaching and guiding role in new anticancer drug research. We hope it will spawn new ideas and new research in the field of anticancer therapy. In what follows, we first create an understanding of why new anticancer drugs are vigorously searched for by highlighting some of the problems associated with a typical chemotherapeutic agent, cisplatin. One possible way to minimise the negative side effects associated with chemotherapeutic agents is to make use of a synthetic polymeric drug delivery system. We explain how such systems may significantly enhance the therapeutic effectiveness of chemotherapeutic agents and highlight some of the structural features they must have. We then develop a general synthetic strategy an experimental chemist may follow to actually synthesise a polymeric drug delivering device and proceed to visualise this strategy utilising a literature example. The need for certain structural features in a delivering device is then motivated by a discussion of the biological mechanism of cell uptake and drug release from the drug carrying device. In conclusion, the synthesis and characterisation of a new example of a polymeric drug delivery system derived from aspartic acid and lysine is presented.

## 2. PROBLEMS ASSOCIATED WITH CHEMOTHERAPEUTIC DRUGS

Many potentially good pharmaceutical agents are either dose limited, or excluded from use in clinical therapy due to insolubility in an aqueous medium, or because of the many severe side effects that they may exhibit. As far as cisplatin, the most successful metal-containing chemotherapeutic drug of recent times [1], is concerned, these side effects include inter alia:

- poor aqueous solubility;
- intensive damage to the linings of the intestines, which leads to the loss of appetite (anorexia) and eventually starving in the case of mice and rats [2];
- severe nausea, vomiting, and audio toxicity [3];
- high toxicity towards the kidneys and bone marrow [4];
- being a moderate carcinogen itself. It can induce lung cancer, skin papillomas, and other carcinomas [5].

In general, most if not all chemotherapeutic drug side effects are due to their general inability to differentiate between diseased and healthy cells [6].

Even if a synthetic chemotherapeutic drug has few side effects, it does not imply success in clinical use. One must realize that chemotherapeutic agents usually are poisons, or at the very least, they are foreign to the body. The defence mechanism of the body, the reticuloendothelial system, recognizes them as such and tries to remove them as fast as possible. A fast excretion rate from the body often proves detrimental to chemotherapy. Cisplatin, for example, is eliminated from the body in a biphasic excretion mechanism in such a way that 50% of the initial administered dose is excreted within 20 hours [7]. After 110 hours, 70% of the initial administered amount of drug is removed from the body by the reticuloendothelial system. The principle mode of excretion in mice appears primarily to be through the urine, 90% of the injected dose being excreted within five days after injection. This quick excretion rate causes large drug concentration fluctuations in the body, and hence, also unpredictable therapeutic activity, a highly undesirable situation. The negative impact of such drug concentration fluctuations in the body becomes apparent when one relates it to 50% lethal dosages (${\text{LD}}_{50}$ values), optimum dosages, and a dosage having no influence on the diseased site at all. For cisplatin [8], the ${\text{LD}}_{50}$ dosage applicable to mice is 14 mg/kg mass of test animal, the optimum dose is half of this, 7 mg/kg test animal, but 3 mg/kg mass of the test animal has absolutely no influence on the diseased site at all. With the excretion profile that cisplatin shows, it is clear that it is extraordinary difficult to maintain drug levels in a patient close to the optimum dose level, but still below the ${\text{LD}}_{50}$ value for prolonged times. The initial required overdose of drug that is required to maintain useful drug levels in the body explains much of the negative side effects of chemotherapeutic drugs.

A further point of consideration in developing and administering anticancer drugs is the development of drug resistance by a tumour after prolonged use. A neoplastic population of cells is not a static entity, but rather an ever-changing one. Tumours display an amazing ability to escape or neutralize the actions of chemotherapeutic agents to which they were initially sensitive [9]. Some common examples of this evasive ability are as follows [10]:

- the loss of specific receptors;
- down-regulation of tumour-associated antigens; and
- shedding of antigens into the body fluids.

The metastatic nature of cancer cells thus leads to the development of drug resistance after continued drug dosage [11, 12].

One of the most fundamental barriers to selective drug delivery involves the so-called targeting problem. The central problem of cancer chemotherapy remains one of selective drug action. Ideally, one would like a toxic agent that can discriminate between neoplastic and nonneoplastic cells. This approach presumes the existence of something to aim at, that is, some molecular characteristic that differs between target and nontarget cells. The metastatic nature of cancer cells makes this a formidable task. Even if a target can be identified, or a cell need can be exploited, as has been done in vitro, the problem remains of actually bringing the pharmaceutical agent, in vivo, from the point of administering to the neoplastic growth. To achieve this, many physiological barriers including the reticuloendothelial system of the body have to be bypassed.

Consequently, to improve the treatment of cancer, research today focusses on developing new and better chemotherapeutic agents having less side effects (i.e., drugs that can zoom onto diseased sites) [13], new methods of drug delivery are being researched [6], combination therapy is being investigated in the hope of finding synergistic effects [14], the scope of radiation therapy is broadened, and completely new methods of combating cancer are sought.

New metal-based drugs include members of the titanocene and ferrocene family of compounds [13, 15]. A promising example of a new method of cancer treatment focusses on photodynamic therapy (PDT) [16, 17]. In PDT, the photo-active drugs in themselves are inactive towards both healthy and cancer cells. However, when light of the correct wavelength is shone on them, the drugs become photo-activated and give rise to conditions that kill the cells it is in contact with, normally via the generation of singlet oxygen. A good measure of selective cancer cell killing is therefore induced into the treatment of a patient merely by focussing light from a suitable laser source onto a tumourous growth. Healthy cells will stay largely undamaged provided that light is not shone upon it. If cancer cells would selectively and preferentially absorb the PDT drug, this would lead to a further enhancement of selective cancer cell destruction. Phthalocyanines and related macrocycles are second-generation PDT agents. Many phthalocyanine derivatives have the advantage of being preferentially absorbed by cancer cells. Some of them were found to be distributed upto 90% selectively in cancer cells, and only 10% in healthy cells after a certain induction period [18].

## 3. SYNTHETIC POLYMERIC DRUG DELIVERY SYSTEMS

Many detrimental properties and side effects of promising anticancer drugs may at least be partially overcome if the pharmaceutical agent is covalently anchored to a polymeric drug carrier. Polymeric or macromolecular drug-carrying devices should be distinguished from sustained drug-release systems. Sustained drug-release systems are normally associated with polymers; frequently insoluble; and in the form of powders, pellets, and capsules from which the slow release of an encapsulated drug from the “container” interior is possible. They normally do not represent systems that may affect target-specific drug delivery as much as they are devices for the continued release of drugs. The term “polymeric drug carrier” describes a polymer or macromolecule that contains covalent *binding sites* that are available to anchor active pharmaceutical units as terminal or pendent groups protruding from the side of the polymeric chain. Polymeric drug-carrying devices may be synthetic or naturally occurring. The naturally occurring ones such as antibodies [19, 20] induce a high mode of selective drug action onto the polymeric drug-containing device, but usually suffer from a lack of large amounts of drug-anchoring sites. Synthetic polymers on the other hand are more prone to suffer from immunogenic side effects [21], but this may be overcome by copolymerisation with ethylene glycol fragments. Their big advantage is that they can be tailor-made to be suitable for almost any purposes and can be engineered to have a large amount of drug-anchoring sites.

The clinical administration of a polymer bound drug, as compared to the free agent, may significantly enhance the therapeutic effectiveness in terms of the following:

- accelerated and unencumbered distribution in the aqueous central circulation system of the body (the blood), thereby reducing the risks of premature degradation and excretion;
- they facilitate cell entry via endocytosis—a cell membrane penetration mechanism generally unavailable to nonpolymeric compounds but highly desired for drugs operating intracellularly;
- more precisely controlled drug serum levels (i.e., the restriction of the drug concentration to the gap between toxic and minimum effective levels);
- an enhanced depot effect through slow drug release from the polymer-drug conjugate.

The structural features of the polymer-drug conjugate required to comply with these attributes include the following:

- a highly flexible linear main chain comprising structural entities that can provide water solubility;
- a sufficiently large molecular mass to prevent quick excretion from the body (the threshold for elimination via the kidneys is ca. 70 000 g ${\text{mol}}^{-1}$);
- a biodegradable carrier backbone prone to catabolic elimination of the “spent” polymer main chain after the payload of drug has been delivered to the target site;
- a large amount of reactive functional groups as suitable binding sites for drug attachment, these sites should be distanced from the main chain by short (5–15 constituent atoms) spacer segments to diminish the steric bulk effect of the polymeric carrier backbone, and they should be sufficiently separated from each other along the backbone to prevent intramolecular multifunctional drug binding;
- the separation of binding sites on the polymeric backbone requires insertion of side chains on the main chain between active binding sites that lack drug-binding capabilities, but enhance other desirable properties. These may include enhancing the hydrophilic nature of the ultimate polymeric drug-carrying device, the incorporation of moieties that enhance drug-targeting capability (i.e., a tumour homing device), antispasmodic properties, or any other desirable physiological effect;
- one or more biofissionable functions (amide, ester, urethane, disulphide, etc.) to be inserted into the carrier-drug connecting link. At least one of these must be sufficiently remote from the main chain to permit enzyme approach and cleavage action to result in effective drug release in a targeted biological compartment;
- carrier backbones and spacers which are non toxic per se. They should especially possess absolute minimal immunogenicity so as to preclude carrier-induced pathological effects. This means polymeric drug-carrying devices should be completely biocompatible.

Akey component towards the success of polymeric drug-carrying devices centres around biodegradable bonds and their capability to home preferentially onto a neoplastic growth. Apart from targeting fleeting receptors on a collection of metastatic neoplastic cells, the nutritional needs of such cells may be utilized to gain preferential neoplastic cell access. Neoplastic cells grow much faster than healthy cells, and have a much higher demand for food in the form of amino acids and energy. Hence copolymers constructed at least partially from amino acids are very attractive as polymeric drug delivery devices. They also introduce the biodegradable amide (peptide) bond. Other degradable bonds include nucleotide and sugar bonds. Sugar groups as a source of energy will especially enhance preferential uptake by neoplastic cells. Other biodegradable groups that may be employed include disulphide bonds that may be reduced to thiol groups [22], or, since cell interior has a lower pH than blood plasma, for example, weakly acid-labile groups such as thio-ethers [23].

## 4. SYNTHETIC STRATEGIES TOWARDS POLYMERIC DRUG DELIVERY SYSTEMS

**Scheme 1:**
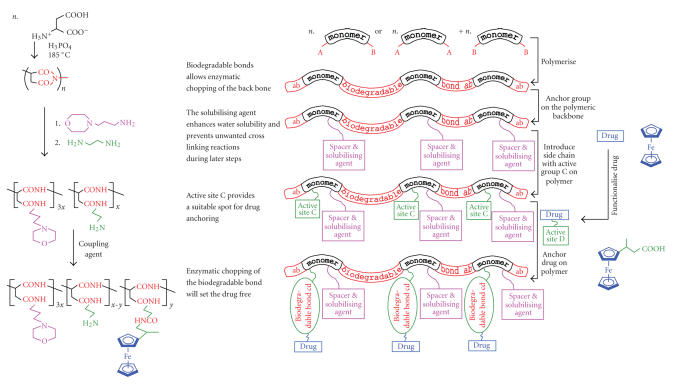
*Main picture*: Colour-coded general synthetic strategy towards the syntheses of polymeric drug carrier devices. Right, in blue: functionalisation of the antineoplastic drug, ferrocene, to 3-ferrocenylbutanoic acid. Left: The synthesis of a specific ferrocene-containing drug delivery device derived from aspartic acid. The colour codes in the side portions are the same as those used in the central main picture. The main picture was reproduced with permission from copyright owner “International Union of Pure and Applied Chemistry.” An adaption of the main part of this scheme was previously published as follows: J. C. Swarts, in *Macromol. Sym*., Eds. K. Levon and A. Guiseppi-Elie), vol 186, pages 123–128, 2002; Copyright Wiley-VCH Verlag GmbH & Co. KgaA, reproduced with permission.

Although models as those mentioned above for synthetic polymeric drug carriers have been in existence for some time [24], until recently only a few efforts were made to actually prepare such compounds. A review by Neuse [25] describes some of the most recent researches in this field. Scheme 1 illustrates the synthetic approach to actually obtain such polymeric drug-delivering devices. The centre of Scheme 1 serves to visualise the overall strategy. At the left and right ends of Scheme 1 an example for an actual synthesis of a polymeric drug delivery system [26] is highlighted next to each colour-coded step of the general strategy. The use of the water-soluble polyaspartic acid drug carrier not only allowed the water-insoluble parent ferrocene-containing drug to be well soluble in water in concentrations exceeding 100 g ${\text{dm}}^{-3}$ but it also increased the cytotoxicity of the active drug as tested on murine EMT-6 cancer cells from an ${\text{LD}}_{90}$ value of 500 *μ*g/${\text{cm}}^{-3}$ in the case of free 3-ferrocenylbutanoic acid to 80 *μ*g/${\text{cm}}^{-3}$ drug content for the polymeric drug delivery device [27].

At least two factors probably contribute to the enhanced activity of the above-mentioned polymeric drug delivery device. The first must be related to the enhanced aqueous solubility of the ferrocene drug. Free ferrocene itself, which is insoluble in water, has been shown to be completely inactive as cytotoxic agent. The second factor centres around the use of aspartic acid as monomeric subunit in the main chain of the polymeric drug carrier. As the cancer cells have a large need for nutrients such as amino acids, the use of a polyamino acid as drug carrier may cause the drug carrying device to be absorbed faster and more efficiently by cancer cells than would be the case for the free drug.

A key factor should be observed in the synthetic sequence shown in Scheme 1. The general strategy in the middle shows the use of difunctional monomers. Aspartic acid is trifunctional. The use of tri- or higher-functional polymerization monomers is useful in the synthesis of polymeric drug carriers, as it provides a free arm where the drug may eventually be anchored. Only two of the three or more monomer functionalities should be used to obtain a linear, highly flexible, and soluble monomer. The other functional group(s) ultimately will provide drug attachment sites. However, normally the use of tri- or higher-functionalised monomers will lead to the formation of highly crosslinked, rigid, and insoluble polymers. In the case of aspartic acid, this did not happen because the formation of stable five-membered succinimide rings is thermodynamically highly favoured. It effectively masked the second carboxylic acid functional group of aspartic acid from unwanted crosslinking reactions. For other tri- or higher-functionalized amino acids, such as trifunctionalised lysine or tetrafunctionalised cystine [22], protection of the additional functional groups is required to prevent formation of pharmaceutically undesired crosslinked polymers.

## 5. MECHANISM OF CELL UPTAKE AND DRUG RELEASE

**Figure 1:**
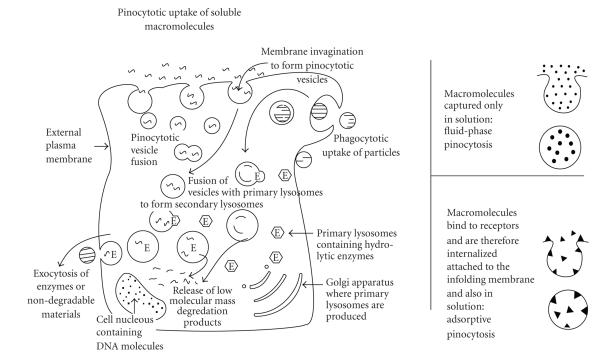
Mechanism of drug uptake and release inside cells. Diagram reproduced with permission from copyright owner “International Union of Pure and Applied Chemistry.” It was previously published as follows: J. C. Swarts, in *Macromol. Sym*., Eds. K. Levon and A. Guiseppi-Elie), vol 186, p123–128, 2002; Copyright Wiley-VCH Verlag GmbH & Co. KgaA, reproduced with permission.

**Scheme 2:**
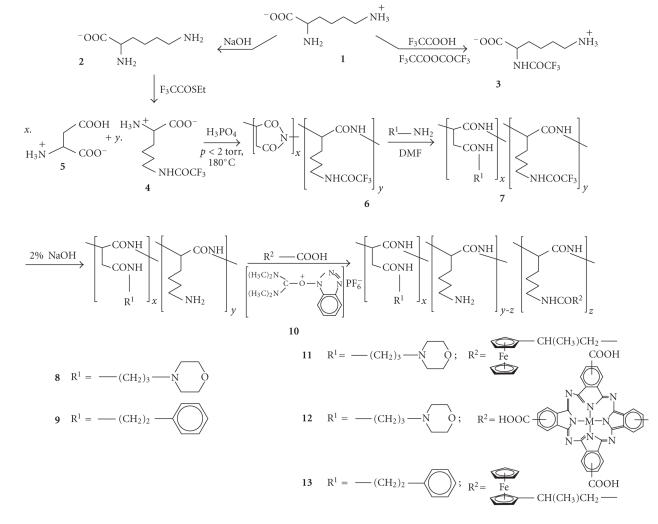
Syntheses of polymeric drug carrying devices **11**, **12** (M = Co), and **13**. For PDT applications, M = Zn or Cl–Al would be more appropriate [18]. All polymers have a random recurring unit arrangement in the backbone. The indicated structures are meant to show building block ratios rather than block polymers. Opening of the succinimide rings of **6** with nucleophiles R^1^-NH_2_ lead to a mixture of *α* and *β* ring-opened aspartates. For convenience, only the *α*-form is shown.

Uptake by neoplastic (and healthy) cells of the soluble polymeric device, shown in Scheme 1, probably occurs by endocytosis, in particular, by fluid-phase pinocytosis as described by Duncan and Kopeček [6]. As shown in Figure 1, polymers in solution are internalised by means of fluid-phase pinocytosis. Cell internalisation may also occur if the polymeric drug delivery device binds to surface cell receptors by means of adsorptive pinocytosis. They are then internalized as attachments to the infolding membrane. Polymeric drug carriers with porphyrins or phthalocyanines attached to them will probably gain access to neoplastic cells by means of adsorptive pinocytosis as this would be consistent with the selective uptake of these macrocycles by cancer cells. Mixtures of fluid-phase and adsorptive pinocytosis may also occur. It was found that pinocytotic uptake by cells of polymers bearing phenol-containing side chains was greatly enhanced [28]. Once internalized, fusion of the pinocytotic vesicles with primary lysosomes takes place to form secondary lysosomes. Release of the drug from the polymeric drug carrier inside the secondary lysosomes occurs by enzymatic hydrolysis of the biodegradable amide bonds that link the drug, ferrocene in Scheme 1, with the polymeric drug carrier, a polyaspartic acid derivative in Scheme 2. Primary lysosomes may contain more than 50 hydrolytic enzymes that are generated in the Golgi apparatus. Next, the drug and other useful low-molecular-mass degradation products like amino acids are released from the secondary lysosomes for use inside the cell nucleous. Finally, the “spent” polymer main chain and any other nondegradable material leave the cell by exocytosis.

A key factor emerges from this discussion. According to this mechanism, no drug is released outside a living cell because these drugs are not in contact with appropriate hydrolytic enzymes. As the folding pattern of the polymeric drug carrier normally inactivates the drug while polymer-bound, it means no negative side effects can be induced by the drug prior to release from the polymeric main chain. In addition, if the polymeric drug delivery device could be designed in such a way that it is internalized selectively into only neoplastic cells, the negative side effects associated with chemotherapy could be circumvented completely.

## 6. THE SYNTHESES OF LYSINE-BASED POLYMERIC DRUG CARRIERS

Lysine, **1**, introduces a new dimension into our discussion of the design of polymeric drug carriers. To prevent crosslinking reactions, one of the three lysine functional groups has to be protected. Here we choose to protect an amino group. One can either protect the *α*- or the *ε*-amino group, Scheme 2, to obtain lysine derivatives **3** or **4**, respectively. Only *α*-amino trifluoroacetyl protected lysine, **3**, is obtained by direct treatment of lysine with trifluoroacetic anhydride in trifluoroacetic acid because the *ε*-amino group is much more basic than the *α*-amino group [29]. The zwitter-ionic structure of lysine, **1**, implies that the *ε*-amino group is mainly in the protonated form and thus well protected against acylation reactions. However, treatment of **1** with ethyl thioltrifluoroactetate under basic conditions [30] according to the method described by Schallenberg gave N^*ε*^-trifluoroacetyllysine, **4**, in 33% yield after recrystallisation from hot water/ethanol in a 2 : 3 ratio. It is advantageous to use the *ε*-amino group rather than the *α*-amino group eventually as the drug-anchoring site because it separates it far enough from the polymer main chain to allow easy enzyme approach to initiate cleavage of amide bonds to facilitate drug release from the eventual drug-carrying devices **11**–**13**. If the *α*-amino group is protected for later drug-anchoring purposes, the steric bulk effect of the polymeric main chain may be so large that enzyme-induced drug release will become kinetically very slow. In addition, it was also previously demonstrated [26] that drug-anchoring reactions of the type shown in Scheme 2 to generate **11**–**13** via amide bond formation proceed more effectively with compounds possessing longer side chains.

We describe here the syntheses, from N^*ε*^-trifluoroacetyllysine **4**, aspartic acid **5**, and polymeric precursors **6** and **7** of polymeric drug carriers **8** and **9**, Scheme 2. We also describe how the ferrocenyl and phthalocyanine moieties may be covalently anchored to the carrier polymers **8** and **9** utilising the coupling agent **10** to obtain the polymeric drug delivery devices **11**, **12**, and **13**.

It was not very easy to predetermine reaction conditions to obtain a specific target *x*/*y* ratio in polymer **6** because of a tendency towards homopolymerisation by aspartic acid. In first experiments, **4** and **5** were thermally copolymerised in a monomer mole ratio of 1 : 10 (**4** : **5**) at $180^{{}^{\circ}}$C under nitrogen and atmospheric pressure in the presence of a polyphosphoric acid (PPA) mass fraction of 0.8 for 8 hours. Two products were isolated. The first was extracted with water, and after dialysis in an 8,000 molecular mass cutoff membrane tubing, **6** was isolated having an *x*/*y* = 2.6/1 ratio, and inherent viscosity $\eta_{\text{inh}}$ = 0.06 dl g^−1^ in 3% yield, m.p = $301^{{}^{\circ}}$C (dec.). The *x*/*y* ratio was established by comparing the ^1^H NMR signal integrals at 5.1–5.5 ppm (CH of imide, one proton per closed imide unit), 4.4–4.6 ppm (CH of aspartic acid, the integral values showed only 65% ring closure of the aspartic acid recurring unit occurred), with that of the combined *β*, *γ*, and *δ* methylene protons of the lysine recurring unit (6 protons per lysine recurring unit) at 0.9–1.8 ppm. The remaining water-insoluble residue was dissolved in DMF and precipitated with ethanol to yield 56% polymer having $\eta_{\text{inh}}$(DMF) = 0.07 dl g^−1^, m.p = $286^{{}^{\circ}}$C (dec.) In this case, the *x*/*y* unit ratio was established by ^1^H NMR as 22/1. The percentage of aspartic acid ring closure was found to be ca. 97%. The remainder of material had molecular mass <8,000 g ${\text{mol}}^{-1}$, and was dialysed away.

When **4** and **5** were thermally co-polymerised in a monomer mole ratio of 1 : 6 = **4** : **5**, other conditions still being the same, the water-soluble fraction was isolated in 8% possessing a monomer ratio of *x*/*y* = 1.4/1. The DMF-soluble fraction was isolated in 30% yield having a monomer ratio of *x*/*y* = 25/1. In another experiment, when the PPA mass fraction was lowered from 0.8 to 0.5, the water-soluble fraction was very small, but the DMF-soluble fraction was isolated in 50% yield having also a monomer ratio of *x*/*y* = 25/1.

The above results showed that **4** and **5** have a tendency to homopolymerise. The low lysine content in each recovered fraction was attributed to the tendency of *α*-amino acids to form 6-membered diketopiperazine rings upon heating [31]. In the case of **4**, this would result in termination of any polymerisation process, and account also for the large % of low-molecular-mass material that was lost upon dialyses. ^13^C NMR spectroscopy showed that the trifluoroacetyl protective group did not survive the polymerisation conditions. However, loss of the trifluoroacetyl protective group must have happened in the latter stages of polymerisation, since no insoluble portion was found in the workup of **6**. Crosslinking was, therefore, ruled out as the cause of the relatively moderate yields.

Further experiments focussed exclusively on a 1 : 2 = **4** : **5** monomer mole ratio in a PPA mass fraction of 0.5 at $180^{{}^{\circ}}$C for only 2.5 hours at pressures below 2 torr. These conditions resulted in almost no water-soluble fraction (<1%), and a DMF-soluble fraction (18% yield, decomposition temperature = $269^{{}^{\circ}}$C) having a repeating unit ratio of *x*/*y* = 7/1 and 29% of aspartic acid fragments still uncyclised. ^13^C NMR showed that the trifluoroacetyl protective group remained intact when using these shorter reaction times. By substituting PPA with 85% ortho H_3_PO_4_, repeated experiments gave products having recurring unit ratios ranging 2/1 < *x*/*y* < 5/1. Yields were slightly higher (25%). The increase in yield and better recurring unit ratios probably arose because it is easier to mix **4** and **5** to a homogeneous distribution in ortho H_3_PO_4_ than in PPA. Lower reaction temperatures ($140^{{}^{\circ}}$C) lowered yields substantially to only 6% and virtually the entire product became water-soluble, even after 7 hours of reaction time. Dialyses were again performed in 8,000 molecular mass membrane tubing. The absence of any ^1^H NMR signals at 5.25 ppm, as well as the presence of the signal at 4.2–4.6 ppm showed that for this product, ring closure of the aspartic acid fragment did not occur. It had a repeating unit ratio of *x*/*y* = 4/1 as compared to the target of 2/1. ^13^C NMR showed that the trifluoroacetyl protective group remained intact under these conditions of decreased temperatures even though the reaction time was increased to 7 hours.

Nucleophilic attack of amines on the succinimide rings of polymeric precursor **6** takes place with ease, and the success rate is independent of *x*/*y* ratios. By choosing the correct nucleophiles, the solubility properties of choice may be introduced to the carrier polymer. Here, the 3-aminopropyl morpholine unit was used to generate water-soluble **8** in 83% yield after stirring the intermediate product **7** with 2% NaOH solution to remove the trifluoroacetyl protecting group and dialysis in 8,000 molecular mass cutoff membrane tubing. Although intermediate **7** can be isolated *en route* to **8**, this is not necessary. One can go directly from **6** to **8** without isolation of 7. 2-Phenylethyl amine was used to generate white, water-insoluble **9** in 90% yield after removal of the protective group and slow precipitation with water from a DMF solution.

To demonstrate the carrier capabilities of **8** and **9**, the antineoplastic ferrocenyl moiety [15] and an example of the family of drugs that may be used in photodynamic cancer therapy [17], a phthalocyanine, have been anchored on these polymeric drug carriers. Both ferrocene and phthalocyanine are insoluble in water when not functionalised, or as carboxylic acid derivatives. Even as sodium carboxylates, they are poorly soluble in water. However, once anchored on the polymeric drug carrier **8**, the ferrocene-containing derivative **11** was soluble in excess of 30 g/${\text{dm}}^{3}$, while the phthalocyanine derivative exceeded an aqueous solubility of ca. 5 g ${\text{dm}}^{-3}$ at pH’s more extreme than those found in the gastrointestinal system (1–8.5) [32, 33]. However, prior to drug anchoring, the ferrocene and phthalocyanine fragments needed to be modified in such a way that coupling with the available amine active sites on the polymeric drug carriers **8** and **9** is possible. To generate a biodegradable bond between drug and polymeric carrier, formation of a peptide (i.e., amide), saccharide, or nucleotide bonds [34] is desirable. Here, the amide bond was aimed for as a biodegradable bond linking carrier to drug. Thus, 3-ferrocenylbutanoic acid [35] and cobaltotetracarboxyphthalocyanine [36] were prepared according to known procedures for later reaction with the amine-containing polymeric carrier devices **8** and **9**.

Although polymers **8** and **9**, the ferrocene-containing derivatives **11** and **13**, and the phthalocyanine-containing derivative **12** are robust, frequently the drug moiety of interest (as opposed to the drug carrier) may be very labile. It is advantageous, therefore, to be able to anchor the drug onto the polymeric carrying device under very mild conditions in order to preserve labile moieties. Also, coupling procedures need to be developed that will allow coupling in media in which both the water-soluble drug carrier, here **8** and **9**, and poorly soluble or aqueous-insoluble drug may be partially dissolved or suspended. Coupling with the aid of O-benzotriazolyl-N,N,N′,N′-tetramethyluronium hexafluorophosphate, **10**, at room temperaturein DMF or DMSO, sometimes with THF or water as cosolvent, satisfies this requirement [26]. 24-hour reactions between tetracarboxyphthalocyanine and **8** with a *x*/*y* ratio of 3/1 in the presence of this coupling agent led to only ca. 10% phthalocyanine binding in **12** as ascertained by ^1^H NMR utilising the phthalocyanine phenyl aromatic proton signal (12 hours per phthalocyanine group) at ca. 7.3 ppm and the –CH_2_–O–CH_2_ morpholine signals (4 hours per morpholine group) at 3.6 ppm. This result means z=0.1y.

The closeness of the COOH functional group in the phthalocyanine moiety had two effects, both associated with an extremely slow coupling rate due to the closeness of the bulky phthalocyanine core (slow coupling reactions because of steric crowding have already been commented on, and are described in reference 25). Firstly, it explains the low binding efficiency (z=0.1y) of cobaltotetracarboxyphthalocyanine to polymeric drug carrier **8**. Secondly, it explains why crosslinking reactions involving cobaltotetracarboxyphthalocyanine apparently did not take place to a noticeable extent. As ascertained by ^1^H NMR, no precipitate from the dialysis tubing other than free cobaltotetracarboxyphthalocyanine (i.e., unbound) could be found.

In contrast, 3-ferrocenylbutanoic acid was anchored to a derivative of **8** having a *x*/*y* ratio of 2/1 ca. 70–80% effectively (i.e., $z_{\text{average}}=0.75y$) within 1 hour at room temperature to give **11** as product. This result was obtained by comparing ^1^H NMR signals (obtained in ${\text{D}}_{2}\text{O}$) of the –CH_2_–O–CH_2_ morpholine signals (4 hours per morpholine group) at 3.53 ppm with those of the CH_3_ substituent in 3-ferrocenylbutanoic acid at 1.1 ppm (it is a well-defined peak that can be uniquely identified next to the lysine signals), the combined *β*, *γ*, and *δ* methylene protons of the lysine recurring unit (6 protons per lysine recurring unit) at 1.2–1.6 ppm, and the aspartate CH protons at 4.2–4.5 ppm. An atomic absorption iron elemental analysis for **11** with *x*/*y* = 2/1 showed an iron content of 4.8% which corresponds to z=0.67y. CHN elemental analyses were not performed.

Coupling of 3-ferrocenylbutanoic acid to a derivative of **9** with *x*/*y* ratio 3/1 to obtain **13** in DMF as solvent was more successful, probably because there was no solvent water present during the coupling reaction, see experimental. By comparing the integral values of the phenyl group ^1^H NMR signals (in DMSO-${\text{d}}_{6-x}$) at 7.15 ppm with those of the CH_3_ substituent in 3-ferrocenylbutanoic acid at 1.2 ppm (it is a well-defined peak that can be uniquely identified between the lysine signals) and the combined *β*, *γ*, and *δ* methylene protons of the lysine recurring unit (6 protons per lysine recurring unit) at 0.9–1.6 ppm, it was ascertained that the ferrocenyl group was anchored 94% efficiently, that is, z=0.94y. An atomic absorption iron elemental analysis for 13 with *x*/*y* = 3/1 showed an iron content of 5.08% which corresponds to z=0.87y. CHN elemental analyses were not performed.

The water-insoluble polymer **12** is of particular interest because it demonstrates two separate functional moieties can be supported on the same polymer drug carrier, here phenyl and ferrocenyl. This begs the question: “can more than one antineoplastic group be anchored on the same polymeric drug carrier?” If this would be possible, combination therapy would be possible without administering more than one type of drug in separate doses to a patient. The synergistic effect usually associated with combination therapy [36, 37] would then be potentially obtainable in a single drug dose by binding to the carrier polymer all the drugs required. This topic is at present being researched. Biological studies are at the moment in progress to determine if present lysine-containing polymeric drug-carrying devices **11** and **12** can compare in efficiency with previously described polyaspartic acid drug-delivering systems [26].

## 7. EXPERIMENTAL

**Equipment and materials**

All organic solvents were distilled prior to use, water was double-distilled. Chemicals were from Merck & CO., Inc. (NJ, USA) or Sigma-Aldrich and used without further purification. Melting points are uncorrected. NMR measurements, at 298 K, were recorded on a Bruker Advance DPX 300 NMR spectrometer. Chemical shifts are reported as *δ* values relative to SiMe_4_ at 0 ppm. Infrared spectra were recorded on a Hitachi 270–50 infrared spectrometer in a KBr matrix. Viscosity measurements were made in a Cannon-Fenske tube at $35.2^{{}^{\circ}}$C in water or DMF. Dialysis was performed in 8,000 molecular mass cutoff cellulose membrane tubing. Iron analyses were performed on a Varian Techtron atomic absorption spectrometer at 272 nm.

**Syntheses**

N^*ε*^-trifluoroacetyllysine, **4**, [30] and cobalto tetracarboxyphthalocynanine [36] were prepared as described before.

A representative example of the synthesis of polymer **6**: Aspartic acid (1.331 g; 10 mmol), N^*ε*^-trifluoroacetyllysine (1.211 g; 5 mmol), and polyphosphoric acid (2.542 g) were thoroughly mixed in a 250 cm^3^ round-bottom flask. The flask was mounted on a rotating evaporator, and lowered (while rotating slowly) into an oil bath preheated to $180^{{}^{\circ}}$. The pressure was reduced after 5 minutes to below 2 torr with the aid of a mechanical pump. After 2.5 hours of slow rotation the reaction mixture was allowed to cool and thoroughly extracted with water. Dialyses of the water extract for 16 hours gave after freeze drying only a negligible quantity of product. The remaining residue after water extraction was dissolved in 20 cm^3^ DMF. Precipitation by ethanol afforded 0.38 g (18%) of **6**; $\eta_{\text{inh}}$(DMF) = 0.08 dl g^−1^, *x*/*y* = 7/1 (see discussion text for conditions to obtain other *x*/*y* ratios); m.p. $295^{{}^{\circ}}$C (dec.). ^1^H NMR (DMSO-${\text{d}}_{6\text{-}x}$): 0.9–1.8 (6H; *β*, *γ* and *δ* CH_2_’s of lysine); 2.1 (3.8 H; CH_2_ of open aspartic acid fragment); 2.6–2.9 (4.8 H; CH of succinimide); 3.0–3.4 (4.8 H; CH of succinimide); 4.4–4.6 (1.9 H; CH of aspartic acid fragment); 5.1–5.5 (4.8 H; CH of succinimide). ^13^C NMR (DMSO-${\text{d}}_{6\text{-}x}$): 110–122, q, CF_3_; 155-156, q, $\underline{\text{CO}}{\text{CF}}_{3}$. IR: 1798, 1720 (C=O); 1219, 1189 (CF_3_).

Polymers **7**, and **9**: A solution of 0.21 g [0.5 mmol, $\eta_{\text{inh}}$ = 0.08 dl g^−1^, *x*/*y* = 3/1] of **6** and 2-phenylethyl amine (0.202 g; 2 mmol) in 5 cm^3^ DMF was stirred for 5 hours at ambient temperature. The newly formed 7 was not isolated (although it can be by precipitation with ethanol or water), but stirred for 5 hours with 20 cm^3^ of a 2% NaOH solution. The resulting clear reaction mixture was dialysed for 16 hours, and freeze dried to give 0.24 g (90% yield) of **9** as a white solid which would not redissolve in pure water, but was soluble in DMSO and DMF; $\eta_{\text{inh}}$(DMF) = 0.07 dl g^−1^; *x*/*y* = 3; m.p $275^{{}^{\circ}}$C (dec.).

Polymer **8**: This polymer was obtained in exactly the same way as polymer **9**, but by replacing 2-phenylethyl amine with 3-aminopropyl morpholine (0.289 g; 2 mmol), and the *x*/*y* ratio of **6** was 2/1. Characterisation data for **8** : yield = 83%; $\eta_{\text{inh}}$ = 0.06 dl g^−1^; *x*/*y* = 2; m.p. $196^{{}^{\circ}}$C (dec.). Unlike **9**, this polymer remained water-soluble after dialysis and freeze drying.

Polymer **11**: To a solution of 0.2 g (0.24 mmol; $\eta_{\text{inh}}$ = 0.06 dl g^−1^; *x*/*y* = 2) of **8** in 1 cm^3^ water was added (in the correct order) 0.035 g (0.35 mmol) triethylamine, a solution of 0.068 g (0.25 mmol) 3-ferrocenylbutanoic acid in 1 cm^3^ THF (the THF may be replaced with DMF) and 0.114 g (0.3 mmol) of O-benzotriazolyl-N,N,N′,N′-tetramethyluronium hexafluorophosphate, **10**. The mixture was stirred for one hour at room temperature during which time the heterogeneous mixture homogenized. Water (10 cm^3^) was added to the reaction mixture before it was centrifuged, dialysed for 16 hours in an 8,000 molecular mas cutoff membrane tubing and freeze dried to give 0.11 g (44%) of **11**; $\eta_{\text{inh}}$ = 0.04 dl g^−1^; x:y:z = 2.00 : 0.35 : 0.65; m.p. $88^{{}^{\circ}}$C, dec. at $105^{{}^{\circ}}$C; Fe anal. 4.8% (req. 4.8% for z=0.65y). ^1^H NMR (D_2_O): 1.0–1.2 (0.65 of 3H, CH_3_); 1.2–1.6 (6H; *β*, *γ* and *δ* CH_2_’s of lysine); 3.53 (8H, CH_2_–O–CH_2_ of morpholine), 3.9–4.2 (0.65 of 9H, C_10_H_9_Fe); 4.2–4.6 (3H, CH of aspartate). IR/cm^−1^: 1660 (C=O); 1550 (NH).

Polymer 12: The same procedure was used as for **11** except that the solvents were all DMF, 3-ferrocenylbutanoic acid was replaced with an equivalent amount of cobalto tetracarboxyphthalocyanine and the reaction time was increased to 24 hours. Characterisation data: 44% yield; $\eta_{\text{inh}}$ = 0.04 dl g^−1^; x:y:z = 2.0 : 0.9 : 0.1; m.p. $194^{{}^{\circ}}$C (dec.); ^1^H NMR (D_2_O): 1.2–1.6 (6H; *β*, *γ* and *δ* CH_2_’s of lysine); 3.53 (8H, CH_2_–O–CH_2_ of morpholine); 4.2–4.6 (3H, CH of aspartate) 7.3–8.3 (0.1 of 12H, phthalocyanine aromatics); IR/cm^−1^: 1720, 1660 (C=O); 1550 (NH).

Polymer **13**: The same procedure was used as for **12**, except that **8** was replaced by an equivalent amount of **9**, tetracarboxyphthalocyanine was replaced with a solution of 0.068 g (0.25 mmol) 3-ferrocenylbutanoic acid dissolved in 1 cm^3^ DMF and the reaction was allowed to proceed for 1 hour only. Characterisation data: 64% yield; $\eta_{\text{inh}}$ = 0.04 dl g^−1^; x:y:z = 3.00 : 0.13 : 0.87; m.p. $180^{{}^{\circ}}$C (dec.); Fe anal. 5.1% (req. 5.1% for z=0.87y. ^1^H NMR (DMSO-${\text{d}}_{6\text{-}x}$): 1.2 (0.87 of 3H, CH_3_); 0.9–1.6 (6H; *β*, *γ* and *δ* CH_2_’s of lysine); 4.1-4.2 (0.87 of 9H, C_10_H_9_Fe); 4.2–4.6 (4H, CH of aspartate). 7.1–7.3 (15 H, C_6_H_5_). IR/cm^−1^: 1655 (C=O); 1553 (NH).

## 8. CONCLUSIONS

In summary, it was shown that upon using the thermal polymerisation technique, aspartic acid and N^*ε*^-trifluoroacetyllysine both have a tendency to homopolymerise. This tendency becomes stronger as reaction conditions become harsher, such as higher temperatures, longer reaction times, or lowering of pressures. Less harsh conditions diminish the tendency to homopolymerisation but also reduce yields. Ring closure of the aspartic acid fragment becomes progressively less efficient when milder reaction conditions are employed. It was also shown that the trifluoroacetyl protective group is less stable at elevated temperatures for prolonged periods of time. Chemical removal of the trifluoroacetyl protective group was achieved under mild alkaline conditions to liberate free amino groups on side chains of lysine-containing potential polymeric drug carriers. Coupling of a carboxylic acid-functionalised ferrocene with the amine-containing polymeric drug carrier was effectively achieved utilising O-benzotriazolyl-N,N,N′,N′-tetramethyluronium hexafluorophosphate as coupling agent. Coupling reactions were more efficient in the absence of water as cosolvent. Coupling of cobaltotetracarboxyphthalocyanine was much less successful. This was presumably due to the poor solubility of cobaltotetracarboxyphthalocyanine in the required solvents.
